# Draft genome assembly for the colombian freshwater bocachico fish, *Prochilodus magdalenae*

**DOI:** 10.3389/fgene.2022.989788

**Published:** 2023-01-19

**Authors:** Jonny Andrés Yepes-Blandón, Chao Bian, María José Benítez-Galeano, Jorge Luis Aristizabal-Regino, Ana Lucía Estrada-Posada, Daiana Mir, Gersson Vásquez-Machado, Víctor Julio Atencio-García, Qiong Shi, Nélida Rodríguez-Osorio

**Affiliations:** ^1^ GIPEN, Piscícola San Silvestre SA, Santander, Colombia; ^2^ Shenzhen Key Lab of Marine Genomics, Guangdong Provincial Key Lab of Molecular Breeding in Marine Economic Animals, BGI Academy of Marine Sciences, BGI Marine, Shenzhen, Guangdong, China; ^3^ Unidad de Genómica y Bioinformática, Departamento de Ciencias Biológicas, CENUR Litoral Norte, Universidad de la República, Salto, Uruguay; ^4^ ISAGEN S.A. E.S.P, Medellín, Colombia; ^5^ HISTOLAB, Bogotá, Colombia; ^6^ FMVZ, DCA, CINPIC, Universidad de Córdoba, Montería, Colombia

**Keywords:** flannel-mouth characiforms, south American fish, colombian bocachico, whole genome sequencing, prochilodontidae

## Abstract

We report the first draft genome assembly for *Prochilodus magdalenae*, the leading representative species of the Prochilodontidae family in Colombia. This 1.2-Gb assembly, with a GC content of 42.0% and a repetitive content of around 31.0%, is in the range of previously reported characid species genomes. Annotation identified 34,725 nuclear genes, and BUSCO completeness value was 94.9%. Gene ontology and primary metabolic pathway annotations indicate similar gene profiles for *P. magdalenae* and the closest species with annotated genomes: blind cave fish (*Astyanax mexicanus*) and red piranha (*Pygocentrus nattereri*). A comparative analysis showed similar genome traits to other characid species. The fully sequenced and annotated mitochondrial genome reproduces the taxonomic classification of *P. magdalenae* and confirms the low mitochondrial genetic divergence inside the *Prochilodus* genus. Phylogenomic analysis, using nuclear single-copy orthologous genes, also confirmed the evolutionary position of the species. This genome assembly provides a high-resolution genetic resource for sustainable *P. magdalenae* management in Colombia and, as the first genome assembly for the Prochilodontidae family, will contribute to fish genomics throughout South America.

## Introduction

The Prochilodontidae (flannel-mouth characiforms) family comprises three phenotypically different genera: *Prochilodus, Semaprochilodus,* and *Ichthyoelephas.* Prochilodontids inhabit several river basins throughout South America. They often form massive populations and can achieve substantial body sizes, making them crucial for subsistence and commercial fisheries (Melo et al., 2016).

The genus *Prochilodus* includes 13 species with a wide distribution in rivers on both sides of the Andes mountains in Colombia, Venezuela, French Guiana, Suriname, Brazil, Peru, Bolivia, *Argentina*, Paraguay, and Uruguay (Castro and Vari 2003). Fishes in this genus carry out reproductive migrations (Kerguelén-Durango and Atencio-García 2015; López-Casas et al., 2016; Lopes et al., 2019), and their life cycles relate to the hydrological patterns of flooding and drought of the swamps and floodplains (López-Casas et al., 2016; Benedito et al., 2018; Lopes et al., 2019).

Five species of the genus *Prochilodu*s inhabit river basins in Colombia: *P. mariae* (Orinoco-river basin), *P. reticulatus* (Catatumbo basin), *P. nigricans* (Amazon-river basin), *P. rubrotaeniatus* (Amazon and Orinoco river basins), and *P. magdalenae* (Magdalena-Cauca, Atrato, and Sinú river basins) (Ortega-Lara et al., 2012; Orozco Berdugo et al., 2014; DoNascimiento et al., 2017).

*Prochilodus magdalenae*, commonly known as “*bocachico*,” is an economically important species for Colombian inland fisheries and an integral part of food security for communities along the Magdalena-Cauca, Atrato, and Sinú river basins. Data compiled in the last 45 years by the Colombian Fisheries Authority (AUNAP) show that *bocachico* catches have decreased dramatically from 40,000 tons in 1975 to around 9,500 tons per year between 2013 and 2016 (Barreto Reyes 2017). Consequently, *P. magdalenae* is now classified as vulnerable (Mojica et al., 2012).

Research on *P. magdalenae* has focused on population ecology, dynamics, and reproduction (Jaramillo-Villa and Jiménez-Segura 2008; Jiménez-Segura et al., 2010). Recent studies based on microsatellite loci and mitochondrial genes have revealed the genetic diversity and structure of *P. magdalenae*, (Aguirre-Pabón et al., 2013; Orozco Berdugo et al., 2014; Landínez-García et al., 2020). Although several nuclear and mitochondrial markers can be used in phylogenetic studies, the lack of complete mitochondrial genome sequences and the genetic similarity between species in the genus*,* has made it difficult to identify the best markers to phylogenetically separate *P. magdalenae* from its closest relative, the Venezuelan *P. reticulatus* (Melo et al., 2018).

Colombia is the second most megadiverse country worldwide, however, its biodiversity is underrepresented at the genetic and genomic levels in widely consulted public databases. Some of the causes for this lack of information include the still high (for the country) cost of NGS technologies and the low National funding for high-throughput molecular research (Noreña et al., 2018).

Considering the importance of bocachico for Colombian fishing and aquaculture and its vulnerable status, better understanding of genetic diversity within this species and evolutionary relationships with sister taxa is critical to effective conservation. The lack of genomic resources for this and closely related species impairs efforts to describe the diversity of this group both between and within species and prevents developing effective domestication and conservation breeding programs. In this work, the first draft reference genome of *P. magdalenae* was produced in support of such studies.

## Materials and methods

### Specimen collection and nucleic acid extraction

All procedures involving the handling of the animals were performed according to the Guide for the Care and Use of Laboratory Animals (Albus 2012) and a permit was granted by the National Aquaculture and Fisheries Authority—AUNAP of Colombia under Resolution 0955 (27 May 2020).

An adult *P. magdalenae* female with 860.0 g body weight, 43.0 cm total length, and species characteristic phenotype was anesthetized with Eugenol (15.0 ml/L), euthanized, and dissected for tissue extraction. High molecular weight genomic DNA was isolated from fresh brain tissue using the QIAGEN MagAttract HMW DNA kit. Samples from brain, gills, heart, stomach, liver, intestine, muscle, and ovary were immediately collected, preserved in RNA*later* ^®^ solution and stored at −20°C until total RNA isolation with the TRIZOL reagent, following the manufacturer’s instructions.

### Genome sequencing and assembly

Sequencing was conducted by Macrogen (South Korea). High molecular weight DNA was divided to generate two independent whole-genome sequencing (WGS) libraries. The first library was prepared with the Illumina DNA Prep kit (Illumina, Inc., San Diego, CA), and was subsequently sequenced using the Illumina Novaseq 6000 platform to generate 150 PE reads. For the second library, genomic DNA was sheared with g-TUBE (Covaris Inc., Woburn, MA, United States) and purified using AMPurePB magnetic beads (Beckman Coulter Inc., Brea, CA, United States). Size-selection was not applied, average sizes were below the 17 kb range with maximum sizes at 20 kb (measured with Bioanalyzer 2100, Agilent). The library was generated from 8 µg of sheared purified genomic DNA, using the SMRTbell Template Prep Kit 1.0 (Pacific Biosciences) and divided on two SMRT PacBio flow cells on the RSII system to generate long sequencing reads.

To estimate genome size we calculated k-mer distribution of the Illumina dataset with Jellyfish 2.2.6 (Marçais and Kingsford 2011), with 17 and 19-mer lengths and used the resulting histograms for genome size calculations, summarized on Supplementary Table S1.

Both raw datasets were first assembled, using MaSuRCA v3.3.5 CABOG assembler, without read preprocessing, as recommended by the software developers (Zimin et al., 2013) using the default settings. Since this was a very fragmented assembly, a second approach for *de novo* genome assembly was carried out, however some further analyses were performed using the MaSuRCA assembly. For the second assembly pipeline, Illumina short reads were filtered with SOAPnuke v.1.5.6 (Chen et al., 2018) with parameters (−l 5 −q 0.5 −n 0.05) and then assembled with Platanus v1.2.1 (Kajitani et al., 2014). Output contigs from this assembly, together with PacBio reads, were further assembled by the DBG2OLC pipeline (Ye et al., 2016) with the following parameters: LD10, MinLen 200, KmerCovTh 6, MinOverlap 80, AdaptiveTh 0.012, and RemoveChimera 1. Subsequently, PacBio reads were mapped onto the previous assembly with minimap2 (Li 2018) with default parameters and the assembly was further corrected with six rounds of Racon v1.2.1 (Vaser et al., 2017). After correction, filtered Illumina reads were mapped onto the corrected assembly with BWA-MEM (Li 2013) and the assembly was further corrected by NextPolish (Hu et al., 2020) with default parameters. Finally the assembled scaffold dataset was compared against the NCBI nucleotide database (May 2022) and the xml file was imported into MEGAN V6.17 (Huson et al., 2016) to rule out contamination.

Repetitive elements in the genome were detected with RepeatModeler v1.0.8 (Smit et al., 2008), TEclass (Abrusán et al., 2009), and LTR-FINDER v1.0.6 (Zhao and Wang 2007) with default parameters to detect and quantify the proportion of repetitive elements. Subsequently, RepeatMasker v4.0.6 (Smit et al., 2013) was used to mask repeated elements in lower case on the draft genome, and build a new library based on the Repbase TE v21.01 (Jurka et al., 2005) upon which repeat elements were discovered in the assembly using RepeatProteinMask v4.0.6. Tandem elements were identified by Tandem Repeats Finder (Benson 1999).

To determine genome representation of the original dataset, trimmed Illumina reads were mapped onto both assemblies with Bowtie2 (Langmead and Salzberg 2012), with the end-to-end option. Heterozygous variations were detected on these mappings with SAMtools (Danecek et al., 2021) and BCFtools.

### RNA-Seq

RNA was isolated using the RNeasy Mini kit (Qiagen. Hilden. Germany), following the manufacturer’s instructions. RNA concentration was measured with the Ribogreen kit (Thermofisher), and RNA integrity was evaluated using an Agilent Technologies 2100 Bioanalyzer. Samples with RIN above seven were used for library generation and sequencing with the TruSeq Stranded mRNA LT Kit (Illumina). Sequencing was performed on a NovaSeq 6000 (Illumina) to produce 150 PE reads.

### Genome annotation

Gene structures were predicted by homology annotation and transcriptome annotation. For homology-based annotation, protein sequences from five representative teleosts, including *Pygocentrus nattereri*, *Astyanax mexicanus*, *Colossoma macropomum*, *Scleropages formosus* and *Danio rerio*, were downloaded from the NCBI database (release 95). These protein sequences were mapped onto our genome assembly by tBLASTn (Gerts et al., 2006) and only those with e-value scores below 10^–5^ were used for the final annotation. Subsequently, gene structures were identified in the dataset with GeneWise v2.2.0 (Birney et al., 2004).

For transcriptome-based annotation, pooled RNA-Seq reads from all sampled tissues were mapped onto the assembly with TopHat2 v2.1.1 (Kim et al., 2013) and gene structures were identified on the RNA-Seq alignment using Cufflinks v2.2.1 (Trapnell et al., 2010). Both gene sets from the above-mentioned approaches were merged by MAKER (Cantarel et al., 2008) to generate a final non-redundant gene set.

Genome completeness was evaluated with BUSCO (University of Geneva Medical School and Swiss Institute of Bioinformatics, Geneva, Switzerland; version 3.03, RRID:SCR_015008) with Actinopterygii_odb9 orthologues database to evaluate the completeness of our assembly.

### Mitochondrial genome curation and annotation

The complete mitochondrial genome scaffold obtained from the MaSuRCA assembly was curated and edited with the CLC Genomics Workbench v20.0.1 (https://digitalinsights.qiagen.com/). The curated genome was further revised by mapping RNA-Seq reads that had successfully mapped onto *P. costatus* mitochondrial genome. Mitochondrial genome annotation was conducted in MITOS (Bernt et al., 2013), using the vertebrate genetic code to contrast to the sequences of annotated mitochondrial genomes in the NCBI (RefSeq 39). Start and stop codons for mitochondrial genes were identified, and transfer and ribosomal RNAs were annotated using structure-based covariance models.

### Phylogenomic analysis

To confirm the assembly fidelity, the final P. magdalenae mitochondrial sequence was used for phylogenomic analysis including five *Prochilodus* mitochondrial genomes (*P. lineatus, P. costatus, P. argenteus, p hartii,* and *P. vimboides*), the mitochondrial genomes of four characid species (*Pygocentrus nattereri, Piaractus brachypomus, Astyanax mexicanus, Psalidodon paranae*), and the zebrafish mitochondrial genome as outgroup. The maximum likelihood (ML) phylogenetic tree was inferred using IQ-TREE v1.6.12 (Nguyen et al., 2015). Branch lengths were estimated with the best fitting nucleotide substitution model (GTR + F + I + G4) according to the Bayesian information criterion scores and weights of the ModelFinder application (Kalyaanamoorthy et al., 2017). Branch support was assessed by the approximate likelihood-ratio test based on the Shimodaira–Hasegawa-like procedure (SH-aLRT) with 1,000 replicates. The tree was midpoint rooted and visualized using the software FigTree v1.4 (Rambaut 2012).

Multiple sequence alignment of amino acid sequences from a representative group of 3,657 single-copy orthologous genes in *Actinopterygii* and one Sarcopterygii (as the outgroup) was performed using MAFFT (Katoh and Standley 2013). TrimAl (Capella-Gutiérrez et al., 2009) was used for the automated removal of poorly aligned regions. A maximum-likelihood phylogenetic tree was inferred using IQ-TREE based on amino acid sequences. Branch lengths were estimated with the JTT nuclear model and branch support with the Ultrafast Bootstrap, and the SH-aLRT procedure with 1,000 replicates. The tree was visualized and edited with FigTree v1.4 and fish silhouettes were obtained from PhyloPic.

### Gene ontology annotation

Functional annotation based on gene ontology (GO) terms was carried out with the non-redundant version of the predicted protein sequences for *P. magdalenae* and protein sequences for *Pygocentrus nattereri* and the *Astyanax mexicanus*. Non-redundant protein sequences were obtained by extracting only the longest transcript for each gene and comparing them with the EggNOG-mapper tool v2.0.0 (Cantalapiedra et al., 2021) based on the orthology assignment method (Trachana et al., 2011). GO terms annotated to the gene models were compared at Gene Ontology level 2 using WEGO genomics 2.0 webserver (Ye et al., 2018). Individual domains (Molecular function, Cellular component, and Biological process) were plotted in R. Terms for each domain with gene number counts of one (1.0) or cero 0) in at least two of the compared species were discarded from the graphical representation. Discarded categories were Obs_chr_num_mai_GO:0090485, for Biological Process; Symplast_GO:0055044, Obs_sub_den_GO:0061618, and Virion_GO:0019012, for Cellular Component; and Tox_act_GO:0090729 for Molecular Function.

### Orthologous gene analysis and biochemical pathway prediction

We used the KEGG Orthology (KO) and KAAS (KEGG Automatic Annotation Server) for ortholog assignment and functional annotation to identify primary metabolic pathways in eukaryotes (Kanehisa 2019). For *P. magdalenae*, we obtained a KEGG pathway annotation for 15,257 global genes and 1,025 genes associated with carbohydrate, energy, lipid, nucleotide, amino acid, glycan, cofactors, and vitamin metabolism. We assessed metabolic pathway completeness, comparing our annotation and the genome annotations of the red piranha and the Mexican tetra*,* generating a heat map graphed with R.

We used SonicParanoid program v1.3 (Cosentino and Iwasaki 2019) to detect single copy and multicopy orthologous groups within the following Actinopterygii species *Prochilodus magdalenae*, *Astyanax mexicanus*, *Pygocentrus nattereri*, *Clupea harengus*, *Danio rerio*, *Ictalurus punctatus*, *Lepisosteus oculatus*, *Scleropages formosus*, *Takifugu rubripes*, and *Xiphophorus maculatus*. We translated the sequences of single-copy orthologous genes to build an amino acid sequence alignment and concatenated the alignments to construct a maximum likelihood tree. Accession numbers for all downloaded genomes are given in Supplementary materials S1.

## Results

### Genome assembly

A total of 442.60 Gb in approximately three billion 150-bp PE reads (> 92% Q30) were obtained from the Illumina library for a coverage of 340X. The PacBio library yielded 22.90 Gb in 2,481,344 long reads (9,230 bp in average length), for 17,6X coverage. K-mer analysis estimated the genome size at 1.3 Gb, which was close to the final assembly size. No significative contamination was detected in the scaffolds.

The first (MaSuRCA) *de novo* assembly produced a 1.3-Gb genome, the GC content was estimated at 42.2% with 29,342 scaffolds (Scaffold N50 = 176,340, average scaffold length = 44,831 bp). The longest scaffold from this assembly was 3,730,485 bp. After filtering for coverage, and length 20,912 scaffolds remained in this assembly. Due to its high fragmentation, a second *de novo* genome assembly was generated (Platanus + DBG2OLC) producing a less fragmented genome with 7,856 scaffolds and a higher scaffold N50 = 348,313 bp.

The average scaffold length for the second assembly was 150,516 bp, and the longest scaffold was 3,963,057 bp. Statistics for both assemblies are summarized in Supplementary Table S2. The second genome assembly was used for most subsequent analyses; however, the complete mitochondrial genome was obtained from the first assembly.

Over 94.0% of Illumina reads mapped successfully to both assemblies, showing a good genome representation of the dataset. These mappings were used for detection of heterozygous variation across the genome. After filtering, 8,872,430 single nucleotide variations (SNV) were detected. The most common substitutions were the transitions G↔A, and T↔C each accounting for 29.5% of the changes, while G↔C transversions represented only 7.3.0%. The transition/transversion ratio was 1.44. A total of 1,266,102 indels were identified, which ranged from 1 to 28 nucleotides. The most common indels involved only one or two nucleotides.

In total, 31.0% of the assembled *P. magdalenae* genome corresponds to repetitive elements (Supplementary Table S3), the majority of which were DNA transposons, followed by LINEs, and LTR elements.

### Genome annotation

Our homology annotation, based on five species, predicted an average of 32,371 gene models with marked differences depending on the species. The number of gene models from the transcriptome-based annotation was higher, leading to a final non-redundant set of 34,725 genes, a considerably higher number than the one expected for the species, according to what has been observed for other *Characiformes*.

A comparison of this genome annotation to the most recent RefSeq annotation reports for *Pygocentrus nattereri* (GCF_015220715.1) and *Astyanax mexicanus* (GCF_000372685.2) shows that the number of genes in this annotation was higher than the values for both close counterparts (See more details about the comparison of these genome annotations in Table 1, and in Supplementary Table S4). BUSCO completeness assessment result for *P. magdalenae* genome annotation detected 94.9% of complete genes, 5.0% duplicated genes, 2.0% of fragmented genes, and 3.1% of missing genes.

**TABLE 1:** Comparative gene annotation metrics for *P. magdalenae* and its closest counterparts red piranha (*Pygocentrus nattereri*) and blind cave fish (*Astyanax mexicanus*), both with chromosome level assemblies and BUSCO completeness values over 95%.

Parameter	*Prochilodus magdalenae*	fPygNat1.pri	*Astyanax*_mexicanus-2.0
		Annotation release 101	Annotation release 102
Gene count	34,725	30,575	30,607
Transcript count	51,264	56,177	49,314
Transcripts/gene	1.4	1.84	1.56
Average transcript length	1,538	3,500	3,006
Longest transcript length	84,798	93,175	86,493
Exon count	575,186	308,137	288,664
Exon average length	175	299	286
Average exons/gene	14.70	10.08	9.11
Average exons/transcript	8.81	5.49	5.85
Intron count	509,862	278,425	258,229
Intron average length	1,581	2,998	2,978
Average introns/gene	13.03	9.11	8.15

### Mitochondrial genome curation and annotation

**FIGURE 1:**
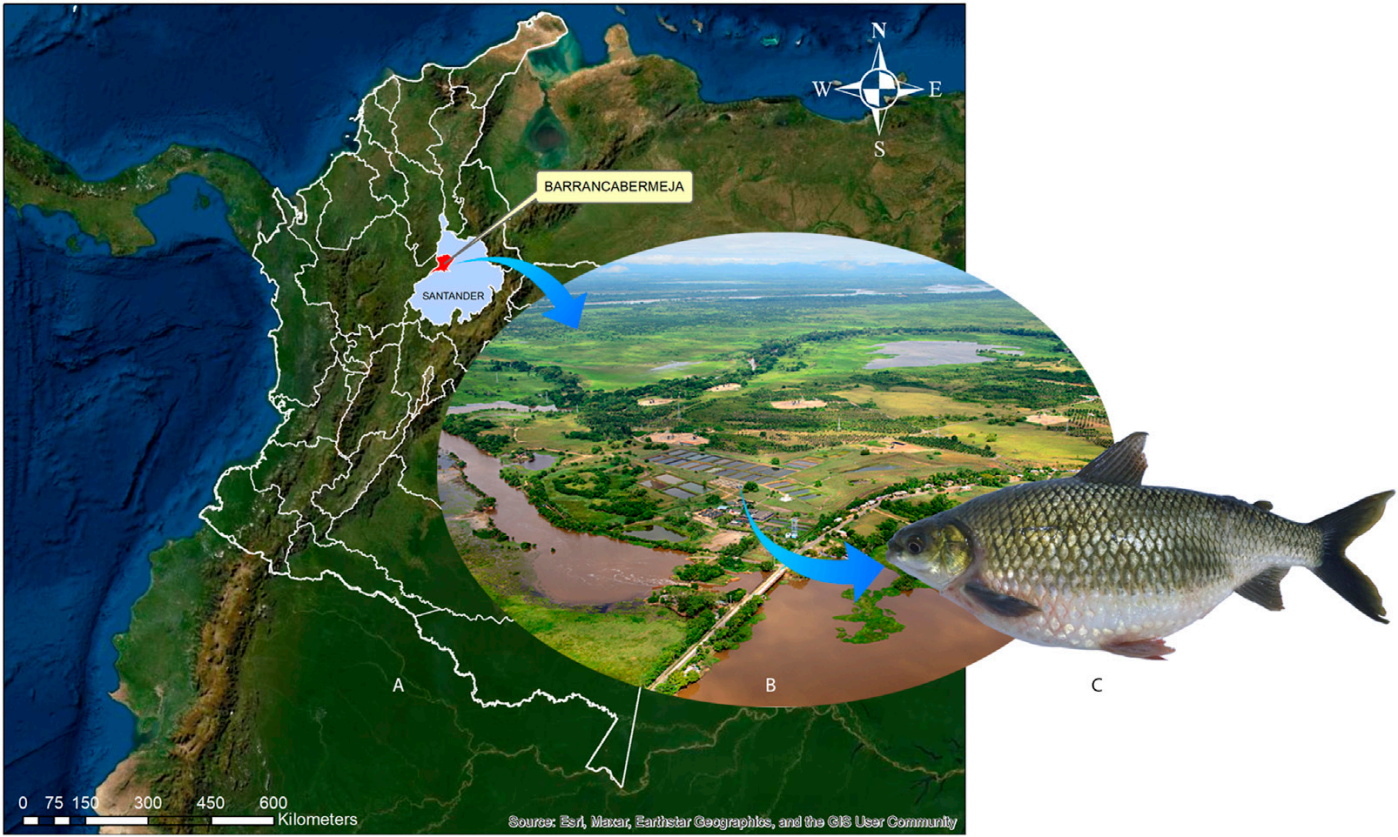
*Prochilodus magdalenae* specimen from which samples were collected and location site.

Two separate scaffolds from the first (MaSuRCA) assembly corresponded to the mitochondrial genome: the first scaffold (38,285 bp) contained a completely duplicated mitochondrial genome (2,3X) in the right orientation; the second scaffold (17,142 bp) contained the mitochondrial genome assembled on the reverse orientation with a partial duplication of the *NAD5* gene. After alignment of both scaffolds the spurious gene duplication was identified and removed. RNA-Seq read mapping confirmed genome orientation. The curated 16,692-bp mitochondrial genome was successfully annotated. Figure 1 is a graphical representation of the mitochondrial genome annotation, showing all 37 genes: 13 genes coding for protein subunits of respiratory complexes (in red), the set of 22 transfer RNAs (in blue), and the mitochondrial small, and large ribosomal RNA subunits 12S rRNA, and 16S rRNA (in green). Supplementary Table S5 provides the detailed mitochondrial genome annotation.

### Phylogenomic analysis

We constructed a Maximum Likelihood phylogenetic tree with our *P. magdalenae* complete mitochondrial genome and five *Prochilodus* species, with complete mitogenome sequences (*P. argenteus, P. harttii, P. costatus, P. lineatus, and P. vimboides*). We also included the mitochondrial genomes of four characids (*Pygocentrus nattereri, Piaractus brachypomus*, *Psalidodon paranae*, and *Astyanax mexicanus*) and the mitogenome of *Danio rerio* (as the outgroup). Supplementary Table S6 contains all the accession numbers for the mitochondrial sequences included in this analysis. The phylogeny constructed with whole mitochondrial sequence corroborated the taxonomic position of *P. magdalenae* in the order and genus.

A comprehensive phylogenomic analysis including 3,697 single-copy orthologous genes from nine representative species in the Actinopterygii class and the sarcopterygian *Latimeria chalumnae* (as the outgroup), agreed with previous analyses for the class and segregated the Otomorpha group with high statistical support (100/100 SH-aLRT) confirming the strong phylogenetic signal of the single-copy orthologous genes. Supplementary Table S7 contains all the accession numbers for the complete genome sequences included in this analysis.

### Orthologous gene analysis and biochemical pathway prediction

Only 71.0% (27,824) of the 39,125 predicted proteins in our annotation had at least one ortholog and 11,301 did not have orthologs. In total 15,257 genes obtained KEGG pathway annotation; of these 1,025 genes were associated with carbohydrate, energy, lipid, nucleotide, amino acid, glycan, cofactors, and vitamin metabolism. In our annotation, 10,177 orthologous groups were present in all tested species, from which 4,222 were single copy groups. Among the multicopy orthologous groups, 109 groups had at least two paralogues in all tested species. For the first cluster of multicopy gene families, 318 are present in *Danio rerio,* while we detected only 168 groups in our *P. magdalenae* annotation.

KEGG biochemical pathway annotation profiles for *P. magdalenae* were comparable to those of *P. nattereri* and *A. mexicanus*, with 1,025, 1,047, and 1,015 annotated enzymes in the primary metabolic processes for each one respectively. Several pathways were fully annotated in all three species, while some pathways lacked one or more blocks in *P. magdalenae* or the other two species. KEGG annotation is summarized in Supplementary Figure S2.

### Gene ontology annotation

Gene ontology (GO) annotation in each category for *P. magdalenae* showed similar profiles and proportions to those of *P. nattereri* and *A. mexicanus*, with the same GO term categories and proportions annotated for all three species in biological process, molecular function, and subcellular localization. *P. magdalenae* annotation showed a higher annotation count, which correlates to the higher number of gene models annotated in this draft. The comparison of GO annotation profiles for the three species is presented in Supplementary Figures S3 and S4.

## Discussion

Despite the high average sequencing depth (> 200X) achieved and the inclusion of high-quality long reads, our first assembly attempt generated a highly fragmented genome with an low N50 metric. A second *de novo* assembly pipeline increased the scaffold length and boosted the scaffold N50 metric two-fold. However, several unresolved regions remain within the scaffolds, probably due to segmental duplications or other complex repeats, which have represented obstacles since the dawn of complex genome assembly (Bailey et al., 2001). Despite the use of long reads, repetitive regions could still be challenging, if coverage is suboptimal (Huddleston et al., 2014; Du and Liang 2019).

Reference-assisted scaffolding was not considered for this assembly, since reference-assisted scaffolding programs yield substantially better scaffolding results when used with closely related reference genomes (Alonge et al., 2019). In this case, the closest species with chromosome-level assemblies are not only outside the *Prochilodus* genus, but out of the Prochilontodidae family. The red-bellied piranha (*Pygocentrus nattereri*) belongs to the Serrasalmidae and the blind cavefish (*Astyanax mexicanus*) to the Characidae. These families are too distant for reference guided assembly to be successful. Different taxonomic and phylogenetic analysis have pointed to a possible earlier separation of the Characidae (with a few other families) from a big clade that included the Serrasalmidae and Prochilodontidae families. The Serrasalmidae then diverged, leaving the Prochilodontidae in the Anostomoidea superfamily, with the Curimatidae, Anostomidae, and Chilodontidae families (Castro and Vari 2004; Sidlauskas and Vari 2008; Guisande et al., 2012; Melo et al., 2016).

Repetitive DNA content in our *P. magdalenae* second assembly was 31.0%, similar to that of its closest relative *Pygocentrus nattereri:* 33.8% (GenBank GCA_015220715.1, RefSeq GCF_015220715.1). However, repetitive DNA content for our first assembly was considerably higher (43.0%), closer to 40.9% which was reported (Warren et al., 2021), for the *Astyanax mexicanus assembly* (GenBank GCA_000372685.2, RefSeq GCF_000372685.2), although lower than that of *Danio rerio* assembly (Howe et al., 2013). This apparent discrepancy in repetitive DNA content between both assemblies could be the result of assembly errors, due to repeats that affected each assembly pipeline differentially.

The low genome contiguity achieved, did not hinder the annotation of coding genes, which showed a similar molecular profile to that of *A. mexicanus* and *P. nattereri* genomes. Almost 95.0% of annotated genes in the assembly were complete, which was close to BUSCO values for *A. mexicanus* (GCF_000372685.2) and *P. nattereri* (GCF_015220715.1) genome annotations, both with chromosome-level assemblies. This level of completeness was similar to other fish genome assemblies (Fernandez-Silva et al., 2018; Kim et al., 2018; Lehmann et al., 2018; Ozerov et al., 2018), which points to a good gene representation in our bocachico genome assembly. However, the higher gene model count obtained in our *P. magdalenae* annotation could still be the result of coding gene fragmentation and spurious gene models generated during annotation.

**FIGURE 2:**
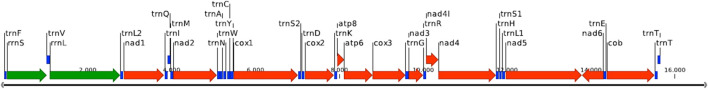
*P. magdalenae* complete mitochondrial genome annotation visualization. In green two ribosomal RNAs, in red 13 protein coding genes and in blue 22 tRNAs.

Our *P. magdalenae* mitochondrial genome confirmed the low mitochondrial divergence within the genus (Melo et al., 2018). The results of mitochondrial and nuclear phylogenies in our analysis are coherent and reproduced the accepted topology for the species. Our phylogenomic tree based on complete mitochondrial sequences (Figure 2), topologically agrees with those built with partial Cytochrome Oxidase C subunit (COI) sequences (Melo et al., 2018). Complete mitogenome phylogenies, as well as those with partial COI sequences, show that *P. vimboides* splits early from the common ancestor of the remaining *Prochilodus* species, followed by bocachico that splits form the ancestor shared by *P. lineatus, P. costatus, P. harttii,* and *P. argenteus*. However, the lack of a complete mitochondrial sequence for *P. reticulatus* prevented us from solving the current phylogenomic ambiguity that places *P. magdalenae* and *P. reticulatus* in the same lineage (Melo et al., 2016; Melo et al., 2018).

Orthologous gene analysis confirmed that over 4,000 detected genes in our *P. magdalenae* annotation have respective orthologs in other Actinopterygii species, and single-copy orthologous genes showed a coherent phylogeny with a tree that reproduced the accepted topology for the species (Supplementary Figure S1). Moreover, the *Characiformes* clade was segregated according to the classification of current higher rank group Otomorpha. The Serrasalmidae and Prochilodontidae families were positioned as sister groups with the Characidae family as basal clade, as reported by morphological and molecular studies (Guisande et al., 2012; Melo et al., 2016).

**FIGURE 3:**
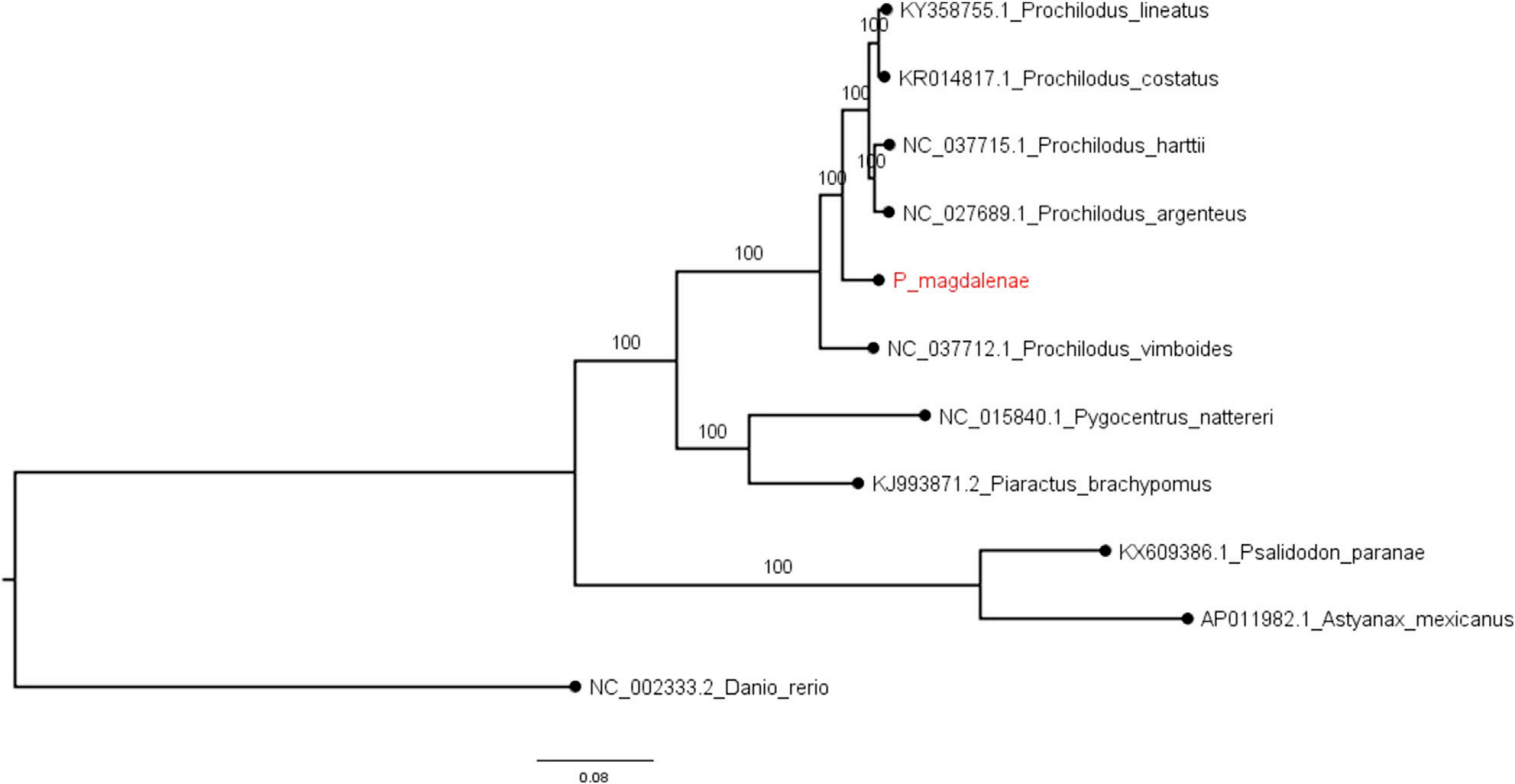
Phylogenomic tree for complete mitochondrial sequences. Examined fishes include five *Prochilodus* species, four characids, and zebrafish (as the outgroup). Node support (aLRT) values are indicated at the nodes. The tree was rooted on midpoint and branch lengths are drawn to scale with the bar at the bottom indicating nucleotide substitutions per site. *P. magdalenae* is highlighted in red.

Primary metabolic pathways and GO annotation showed remarkably similar gene annotation profiles for *P. magdalenae*, *Pygocentrus nattereri* and *Astyanax mexicanus*. However, the tryptophan metabolic pathway was incomplete in our annotation. The crucial role of this amino acid in protein and serotonin synthesis (Höglund et al., 2019) might hint at a limitation in this annotation version, that should be revised in the future. (Figure 3) Further work is needed to complete the annotation in this species and understand the functional genomics implications of differences with its sister species.

## Conclusion

This genome assembly not only represents a high-resolution genetic resource for sustainable bocachico management, but, as the first draft genome for the *Prochilodus* genus and the Prochilodontidae family, it is a valuable contribution for flannel-mouthed characins genomics. Despite the elevated fragmentation, the high gene completeness achieved here is an indicator of the potential of this draft genome to be used in genomic and transcriptomic studies. Further assembly and annotation efforts are necessary to increase genome contiguity for bocachico and to improve the annotation limitations found in this draft.

## Data Availability

The datasets presented in this study can be found in online repositories. The names of the repository/repositories and accession number(s) can be found below: https://www.ncbi.nlm.nih.gov/, PRJNA848980.
