# Supplementary material for: Draft genome assembly for the colombian freshwater bocachico fish, Prochilodus magdalenae
Source: Front Genet. 2023 Jan 19;13:989788. doi: 10.3389/fgene.2022.989788 (PMC9893009; doi:10.3389/fgene.2022.989788)
Supplement: Supplementary file 1 [file Table1.pdf]

SUPPLEMENTARY TABLES

Supplementary Table 1: Summary of k-mer distribution for genome size estimation.

k-mer	K-mer num	K-mer depth	Genome size	Used bases	Used reads
17	98,927,272,620	75	1,319,030,302	110,651,986,412	732,794,612
19	97,461,683,396	73	1,335,091,553	110,651,986,412	732,794,612

Supplementary Table 2: Metrics for both genome assembly approaches

Parameters	MaSuRCA Assembly 1	Platanus + DBG2OLC Assembly 2
**Total sequence length (bp)**	1,303,576,362	1,182,454,182
**Total number of scaffolds**	29,342	7,856
**Average scaffold length (bp)**	44,831	150,516
**Largest scaffold (bp)**	3,730,485	3,963,057
**N25 stats: (bp)**	571,686	760,858
**N50 stats: (bp)**	176,340	348,313
**N75 stats: (bp)**	54,024	142,039
**L50 stats (#)**	1,433	844
**L95 stats (#)**	13,735	5,355
**Total GC count: (bp)**	555,315,698	499,586,892
**GC %:**	42.21 %	42.25%

Supplementary Table 3: Classification of repeat sequences in the assembled genome**.**

Type	Combined TEs	
	**Length (bp)**	**% in genome**
Denovo	203,212,986	17.2
Repeatmasker	93,065	0.008
Proteinmask	54,874,156	4.6
Trf	143,460,375	12.1
**Total**	**365,710,384**	**30.928**

Supplementary Table 4: Statistics for predicted protein-coding genes.

**Type of annotation**	**Species**	**Gene Number**
Homology- based	*D. rerio*	41,080
	A. mexicanus	16,413
	C. macropomum	37,563
	P. nattereri	40,950
	S. formosus	25,848
Transcriptome-based		51,264
**Final annotation (MAKER)**		**34,725**

Supplementary Table 5: MITOS output for *Prochilodus magdalenae* mitochondrial genome annotation

**Name**	**Start**	**Stop**	**Strand**	**Length**
**trnF(ttc)**	1	68	+	68
**rrnS**	69	1020	+	952
**trnV(gta)**	1020	1091	+	72
**rrnL**	1092	2770	+	1679
**trnL2(tta)**	2771	2845	+	75
**nad1**	2861	3814	+	954
**trnI(atc)**	3829	3900	+	72
**trnQ(caa)**	3899	3969	-	71
**trnM(atg)**	3969	4037	+	69
**nad2**	4038	5075	+	1038
**trnW(tga)**	5083	5154	+	72
**trnA(gca)**	5157	5225	-	69
**trnN(aac)**	5227	5299	-	73
**trnC(tgc)**	5331	5397	-	67
**trnY(tac)**	5399	5469	-	71
**cox1**	5477	7009	+	1533
**trnS2(tca)**	7019	7089	-	71
**trnD(gac)**	7095	7167	+	73
**cox2**	7182	7865	+	684
**trnK(aaa)**	7873	7947	+	75
**atp8**	7949	8113	+	165
**atp6**	8107	8787	+	681
**cox3**	8790	9572	+	783
**trnG(gga)**	9575	9646	+	72
**nad3**	9647	9994	+	348
**trnR(cga)**	9996	10065	+	70
**nad4l**	10066	10359	+	294
**nad4**	10356	11729	+	1374
**trnH(cac)**	11737	11805	+	69
**trnS1(agc)**	11806	11873	+	68
**trnL1(cta)**	11875	11947	+	73
**nad5**	11966	13777	+	1812
**nad6**	13786	14301	-	516
**trnE(gaa)**	14302	14370	-	69
**cob**	14376	15509	+	1134
**trnT(aca)**	15517	15588	+	72
**trnP(cca)**	15588	15657	-	70

Supplementary Table 6: Species included in complete mitochondrial genome phylogenomic analysis

Species	Accession Number	Length (bp)
Astyanax mexicanus	AP011982.1	16,682
Psalidodon paranae	KX609386.1	16,707
Danio rerio	NC_002333.2	16,596
Piaractus brachypomus	KJ993871.2	16,722
Prochilodus argenteus	NC_027689.1	16,697
Prochilodus costatus	KR014817.1	16,699
Prochilodus harttii	NC_037715.1	16,697
Prochilodus lineatus	KY358755.1	16,698
Prochilodus vimboides	NC_037712.1	16,696
Pygocentrus nattereri	NC_015840.1	16,706

Supplementary Table 7: Species included in single-copy orthologous comprehensive phylogenomic analysis

Species	Accession Number
Astyanax mexicanus	GCA_000372685.2
Clupea harengus	GCA_900700415.1
Danio rerio	GCA_000002035.4
Ictalurus punctatus	GCA_001660625.1
Latimeria chalumnae	GCA_000225785.1
Lepisosteus oculatus	GCA_000242695.1
Pygocentrus nattereri	GCA_001682695.1
Scleropages formosus	GCA_900964775.1
Takifugu rubripes	GCA_901000725.2
Xiphophorus maculatus	GCA_002775205.2
