# Supplementary material for: Draft genome assembly for the colombian freshwater bocachico fish, Prochilodus magdalenae
Source: Front Genet. 2023 Jan 19;13:989788. doi: 10.3389/fgene.2022.989788 (PMC9893009; doi:10.3389/fgene.2022.989788)
Supplement: Supplementary file 2 [file Image1.pdf]

SUPPLEMENTARY FIGURES

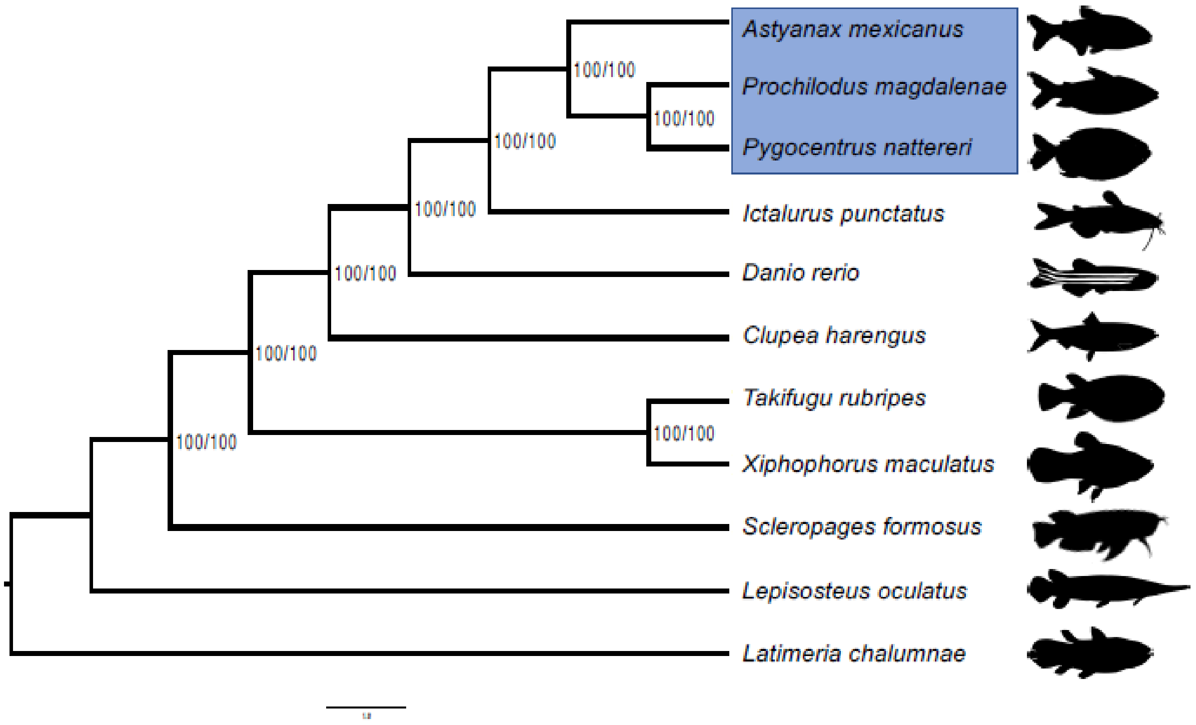

Supplementary Figure 1. Phylogenomic tree for of 3.657 single-copy orthologous genes from nine representative species in the Actinopterygii class and the sarcopterygian *Latimeria chalumnae* (as the outgroup). In the blue box, *P magdalenae* and its closely related species *Astyanax mexicanus* and *Pygocentrus nattereri*

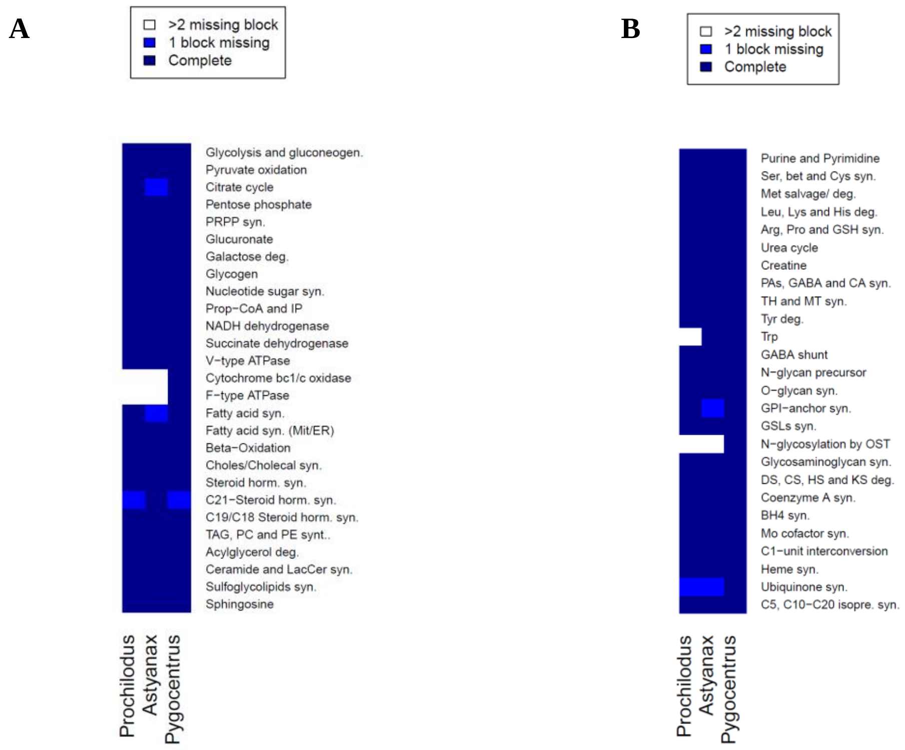

Supplementary Figure 2 KEGG biochemical pathway annotation profiles for *P. magdalenae Pygocentrus. nattereri* and *Astyanax_mexicanus.*

A. Main carbohydrate, energy, and lipid metabolism

B. Main nucleotide, amino acid, glycan, glycosaminoglycan, and vitamin metabolism

Abbreviations: A: Gluconeogen: gluconeogenesis, syn: biosynthesis, deg: degradation, Prop-CoA: Propanoyl-CoA, IP: Inositol phosphate, Mit: Mitochondrial, ER: endoplasmic reticulum, Choles: Cholesterol, Choleca: Cholecalciferol, horm: hormone, TAG: Triacylglycerol, PC: Phosphatidylcholine, PE: Phosphatidylethanolamine, LacCer: Lactosylceramide.

B: syn: Biosynthesis, deg: degradation, Ser: Serine, bet: Betaine, Cys: Cysteine, Met: Methionine, Leu: Leucine, Lys: Lysine, His: histidine, ARG: Arginine, Pro: Proline, GSH: Glutathione, PAs: Polyamine, CA: Catecholamine, TH: Thyroid hormone, MT: Melatonin, Tyr: Tyrosine, Trp: Tryptophan, GSLs: Glycosphingolipid, OST: oligosaccharyltransferase, DS: Dermatan Sulfate, CS: Chondroitin sulfate: HS: Heparan sulfate, KS: Keratan sulfate, BH4: Tetrahydrobiopterin, Mo: Molybdenum, Isopre: Isoprenoid.

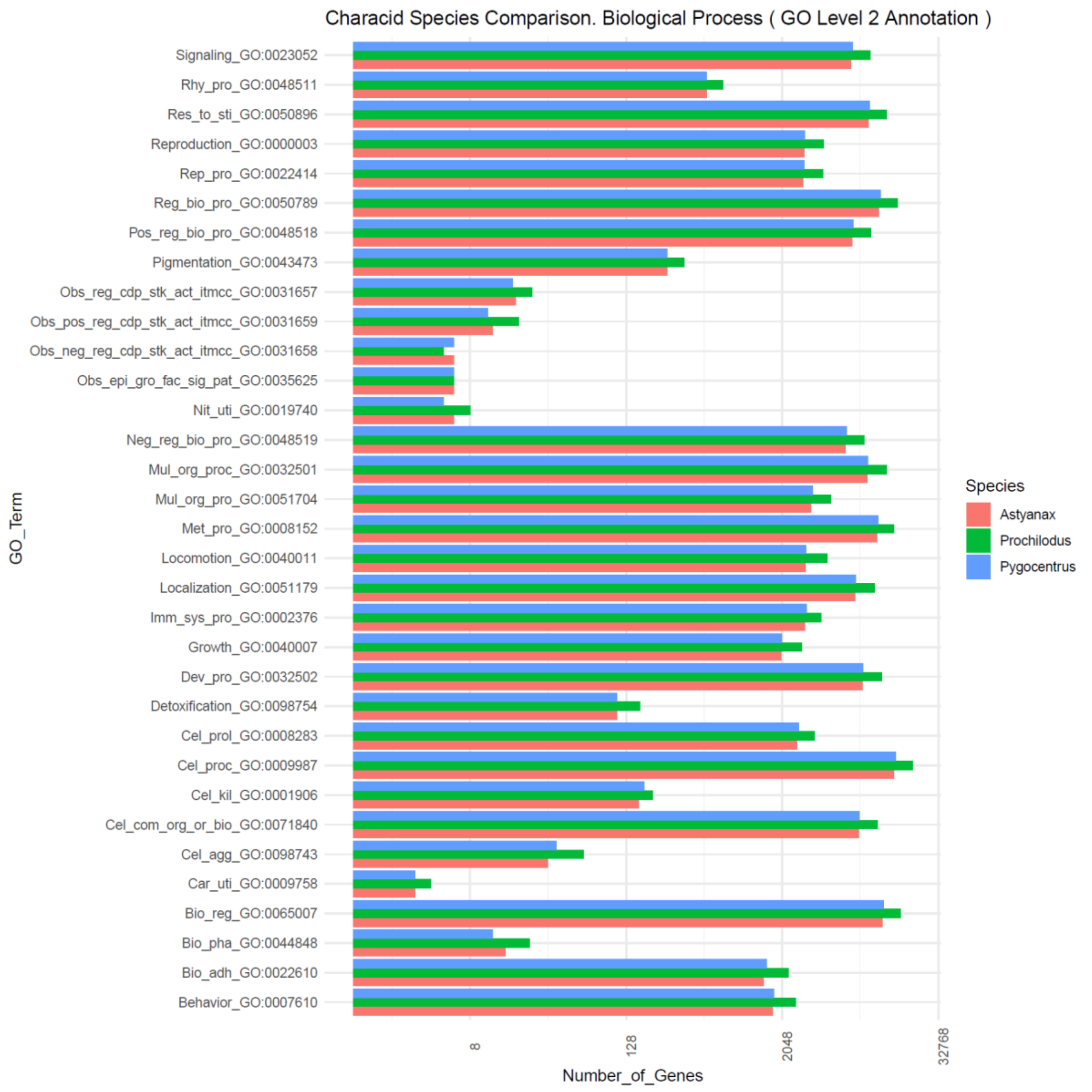

Supplementary Figure 3 GO Level 2 Biological process term frequencies for *Astyanax mexicanus* (137,066 terms), *Prochilodus magdalenae* (191,617 terms), and *Pygocentrus nattereri* (140,135 terms). The X-axis represents log2 base transformed term count

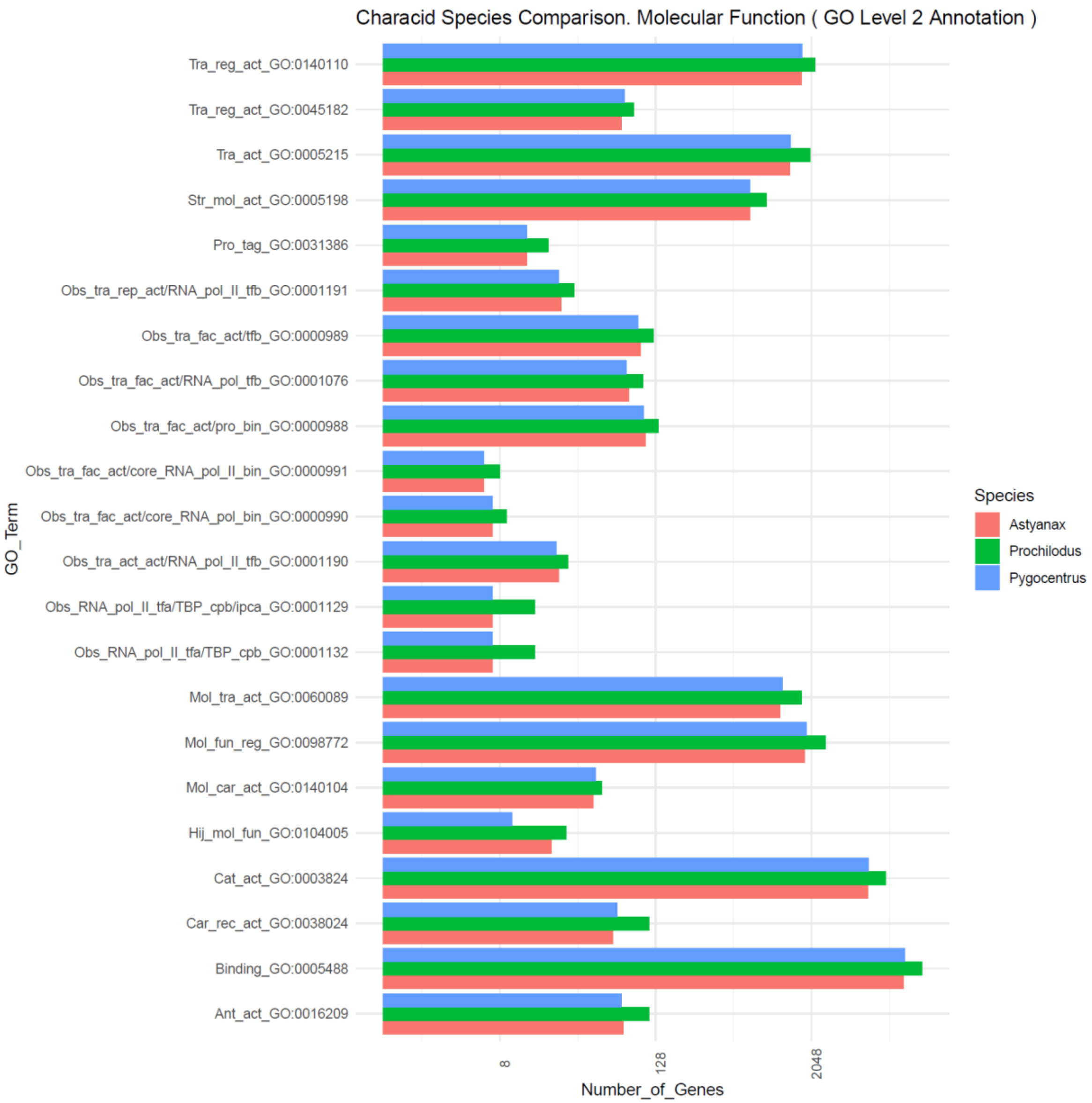

Supplementary Figure 4 GO Level 2 Molecular Function term frequencies for *Astyanax mexicanus* (23,485 terms)*, Prochilodus magdalenae* (32,627 terms), and *Pygocentrus nattereri* (23,967 terms). The X-axis represents log2 base transformed term count

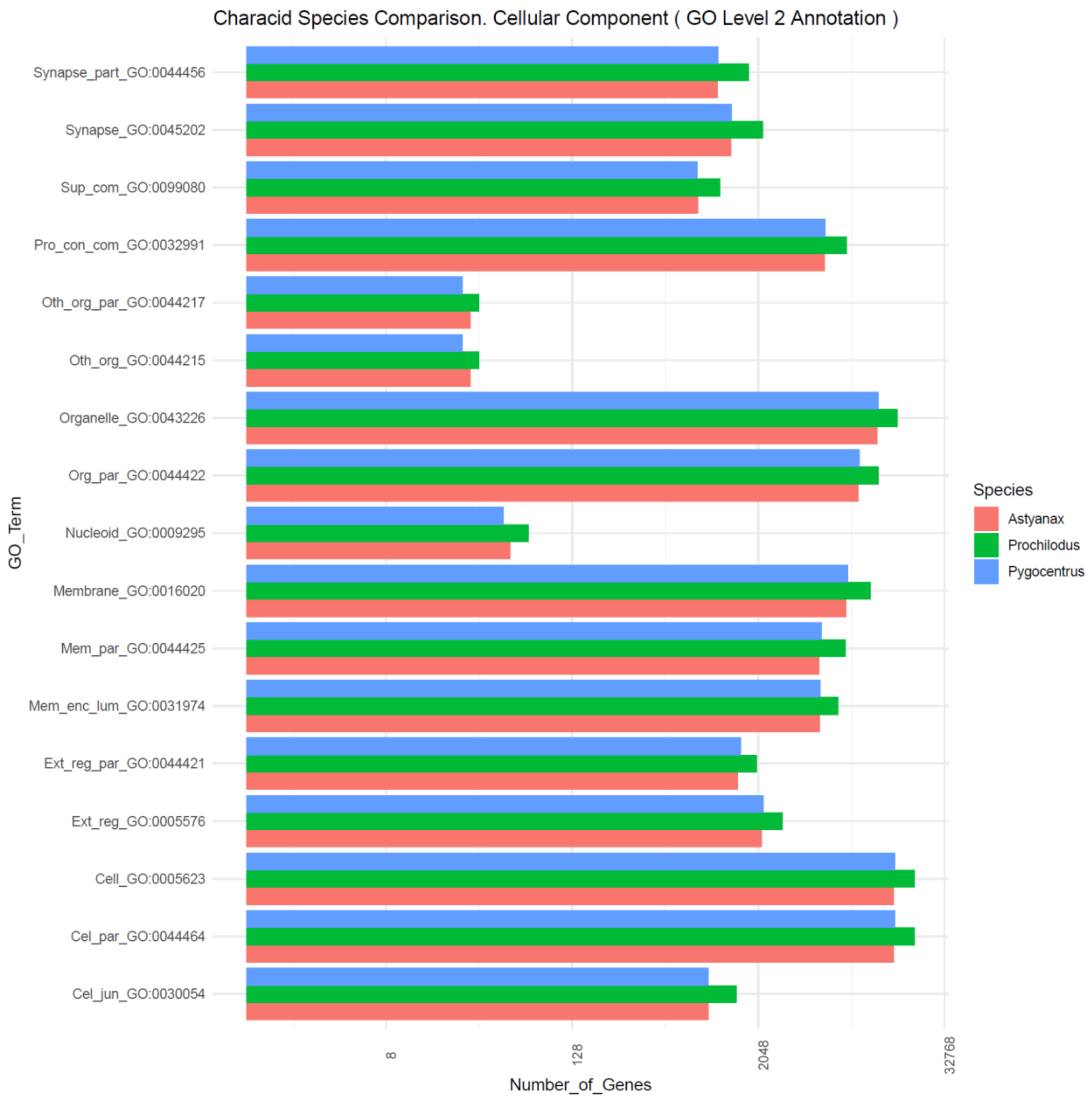

Supplementary Figure 5 GO Level 2 Cellular Component term frequencies for *Astyanax mexicanus* (83,084 terms), *Prochilodus magdalenae* (114,715 terms), and *Pygocentrus nattereri* (84,476 terms). The X-axis represents log2 base transformed term count
